# Transcription Factor DLX5 As a New Target for Promising Antitumor Agents

**Published:** 2011

**Authors:** R.A. Timakhov, P.O. Fedichev, A.A. Vinnik, J.R. Testa, O.O. Favorova

**Affiliations:** Quantum Pharmaceuticals, Russia; Pirogov Russian National Research Medical University; Fox Chase Cancer Centre, Philadelphia, USA

**Keywords:** DLX5, transcription factor, small molecules, cancer, molecular docking

## Abstract

The crystal structure of the human transcription factor DLX5 has been used for the screening of a library consisting of 10^6 ^compounds by the molecular docking technique.*In vitro *tests**of the 14 top-rated ligands showed that compound Q12 displays the best ability to inhibit the proliferation of*Dlx5 *positive mouse lymphoma cells, which correlates with the down-regulation of*c-myc*expression. Compound Q12 has low toxicity on normal human ovarian epithelial cells and mouse lymphoma cells with absent expression of*Dlx5*, and can be used for further chemical optimization and for the development of novel, highly efficient cancer treatments.

## INTroduction

A wide range of drugs have been used in modern clinical practice in order to control cancer [1­­–3]. However, even if all the available drugs were to be used, the proportion of patients who respond to the therapy would remain rather small. For this reason is critically necessary to design new efficient targeted methods for cancer treatment based on a deep comprehension of the mechanisms of tumor growth.

Recent discoveries reveal that the transcription factor DLX5 displays oncogenic activity. The overexpression of the *DLX5* gene in mammalian cells stimulates cell proliferation [4] and can be observed in endometrial carcinoma, non-small cell lung cancer (NSCLC), and small cell lung cancer [5, 6]. The knockdown of the *DLX5* expression using siRNA in mouse and human cancer cells results in the arrest of cell proliferation [4, 7]. New data point to the fact that DLX5 has a direct effect on the expression of protooncogene *c-myc* [8]. All these facts allow us to regard DLX5 as a promising target for which specific ligands that have the properties of oncogenesis inhibitors can be found.

Attempts have frequently been made to use the so-called “high throughput screening” to solve the problem of the search for the ligands of a certain protein [9–13]. This screening is carried out on a cell culture or on an *in vitro * model, using an earlier prepared compound library. The logistics and cost of the studies required for the experimental validation of a significant number of molecules is prohibitively high in many cases. On account of these reasons, in the present study we used the algorithm earlier elaborated to search for inhibitors of new protein targets, based on the analysis of the crystal structure of a target protein [14]. The algorithm is based on the molecular docking of chemical compounds to the known 3D model of a target protein, which predicts the possible position of a compound in the protein–ligand binding site, the calculation of the molecular dynamics being used to refine the binding energies for the best suiting compounds. As shown in our study, as well as in previous studies [14–19], this multi-level approach is not only efficient, but it also considerably reduces the amount of experiments to be carried out. In this case, it enabled the discovery of several ligands of the transcription factor DLX5 that have potential for cancer therapy.

## EXPERimental

**Ligand preparation and molecular docking**

In order to optimize the time of computational screening, the ENAMINE library consisting of 10 ^6 ^ compounds was clustered using the Jarvis–Patrick algorithm [20, 21] with acceleration [22], which is contained in the QUANTUM software package. The so-called Tanimoto metric was calculated using the Daylight molecular fingerprints, which were selected as the measure of molecular similarity [23]. The parameters of clusterization were selected in such a manner that each cluster consisted, on average, of approximately 10  related structures; the total number of non-clustered molecules being no higher than 20% of the initial amount of the library compounds. The compounds representing the centroids of clusters were then selected for further screening. In order to enhance the speed of molecular docking, from the entire centroid library were selected the molecules with the low molecular weight. All the selected compounds were extracted from the sdf files provided by ENAMINE and processed in the batch-mode. The library had not been additionally enriched with molecules active towards oncotargets or by any other methods. The typization of protein and ligands, as well as *in silico * screening, was carried out using the corresponding tools from the QUANTUM software package.

The software predicts the binding (affinity) constants ( *K*_d_ ) between small molecules and a target protein with an accuracy of approximately one order of magnitude, through the estimation of their intermolecular interactions, by using accurate models of atomic forces in an aqueous environment [14, 24–26]. The hierarchy of physical models of intermolecular interactions was used for calculations. In order to initially find the position of a ligand in the active site of a protein and estimate the binding energy of the protein–ligand complex, docking of the ligand to the rigid protein structure was carried out; the results were then refined using the flexible protein model. The modified model of inter and intramolecular interactions AMBER/GAFF were used [27] in order to estimate the potential energy of interaction. The free energy was estimated using the linear interaction model [28]. An aqueous environment was simulated using the modified generalized Born model [25]. The algorithm was described earlier [14], where it was used to search for the inhibitors of protein–protein interactions.

Molecular docking of the molecules from the initial compound library was performed to the rigid structure of the DLX5 2DJN protein obtained from the Protein Data Bank (PDB) [29]. The region of the 3D structure that was selected for molecular docking was 2 x 2 x 2 nm in size. The ligands with the best predicted binding energies were recalculated to the models with a flexible protein [14, 15]. In order to verify that the selected molecules had not been described earlier, the following facts were checked: whether or not these compounds and/or their analogues had been contained in the database or had been mentioned in the reviews devoted to the known inhibitors.

**Cell cultures**

In the present study we used the earlier characterized [30] line 42 of T-cell lymphoma from Akt2-transgenic mice (42-936, 42-577, and 42-588) and line 72 (wtl36). The cells were incubated in the Iscove’s MDM medium containing 10% of fetal bovine serum (FBS).The other cell lines were incubated in the RPMI medium with 10% of FBS. All cell cultures were kept at 37 ^0^ С at an atmosphere of 5% CO _2_ . Potential inhibitors of DLX5 (Dlx5) were added to the medium containing 10 ^5^ tumor cells at a concentration of 10 µM followed by incubation for 96 h at 37 ^0^ С. The cell proliferation was assessed using the CellTiter 96 Aqueous One Solution Assay (Promega) in accordance with the manufacturer’s protocol. Each experiment was repeated at least three times.

**RNA extraction and real-time RT-PCR**

The RNA was extracted from the line 42 mouse T-cell lymphoma cells after incubation with compound Q12 and DMSO for 96 h using the RNAqueous® Kit, in accordance with the manufacturer’s protocol. Real-time RT-PCR (repeated at least in triplicates) was carried out in a specialized service of the Fox Chase Cancer Center. The samples for estimation of the expression of the *c-myc, Dlx5* , and *Tbp * genes were synthesized at Applied Biosystems.

## RESULTS AND DISCUSSION

**Fig. 1:**
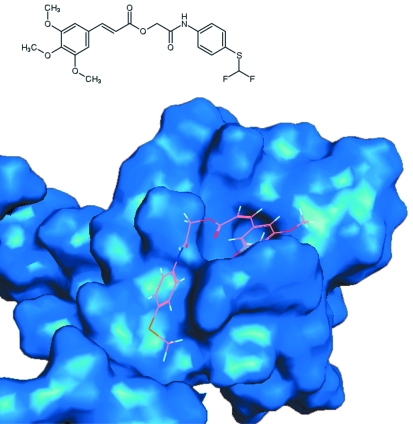
Molecular docking of one compound selected for further experiments: chemical structure and position of compound in the active site the transcription factor DLX5.

Screening for the new ligand molecules specific to DLX5 has been carried out with The QUANTUM software suite, based on the analysis of the protein crystal structure [14, 24–26]. This approach not only enables the identification of the molecules with potential to bind with a certain protein, but also allows us to minimize the quantity of false positive results, when the molecules with a high binding energy predicted *in* *silico * manifested no functional activity in the experiment. The search was made more complicated by the absence of preliminary data on the binding of the known compounds with DLX5 protein; therefore, blind studies were performed. The best molecules and all their structural analogues from the original ENAMINE library were sorted on the basis of their predicted binding energy. According to the results of molecular docking, 100 ligands were selected; 14 of those with the best predicted binding energy of DLX5 protein were ordered and synthesized at ENAMINE company; then, they were tested on cell cultures. *Fig. 1* shows an example of molecular docking with an active DLX5 site of one of these ligands.

**Fig. 2:**
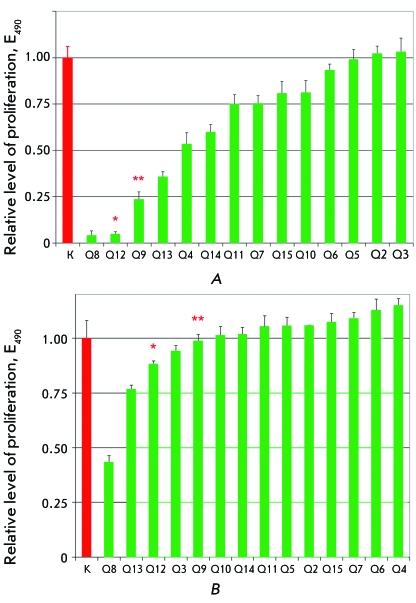
Experimental estimation of the properties of 14 compounds, for which high binding energy for the 3D structure of DLX5 was predicted: their efficacy was estimated by the impact on proliferation of Dlx5 positive mouse lymphoma cells 42-936 (A) and cytotoxicity measured by the impact on proliferation of normal human ovarian epithelial cells (B). K - is the level of proliferation in the control. The columns depicting the effect of the most promising compounds are labeled with asterisks (Q12(*) and Q9(**)).

The cells of the earlier characterized line 42 of T-cell lymphoma from Akt2-transgenic mice [4, 30] were used as a model to verify the specific activity of the selected ligands. These cells bear a clonal chromosome rearrangement –chromosome 6 inversion, which results in the translocation of the *Dlx5 * gene into the region under control of the T-cell enhancer and in the overexpression of Dlx5 protein. Lymphoma cells 42-936 were incubated with each of the 14 selected DLX5 ligands; the impact of the ligands on proliferation was assessed. As can be seen in *Fig. 2A* , the ligands demonstrate different efficacies of impact on the proliferation of lymphoma cells; compounds Q8, Q12, Q9, and Q13 manifested the best inhibitory activity. The possible nonspecific cyto-toxic action of the selected compounds was tested on normal human ovarian epithelial cells without DLX5 expression ( *Fig. 2B* ). When comparing with the control, it can be seen that most ligand molecules, with the exception of the compounds Q8 and Q13, manifest no significant cyto-toxicity. Since Q8 and Q13 manifested a cytotoxic effect, they were eliminated from further consideration. Compounds Q12 and Q9 were selected for further studies as the most promising ones.

**Fig. 3:**
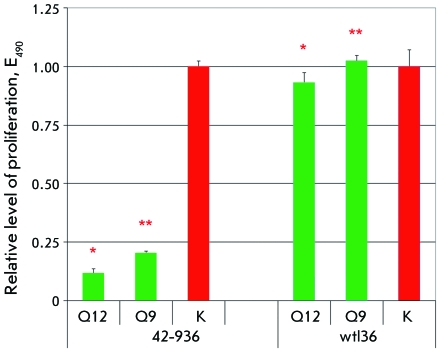
The selectivity of compounds Q12 and Q9 on mouse lymphoma cells. Left panel: the impact of compounds on proliferation of Dlx5-positive mouse lymphoma cells 42-936. Right panel: the impact of compounds on proliferation of Dlx5-negative mouse lymphoma cells wtl36. Additional labeling is identical to that in *Fig. 2.*

**Fig. 4:**
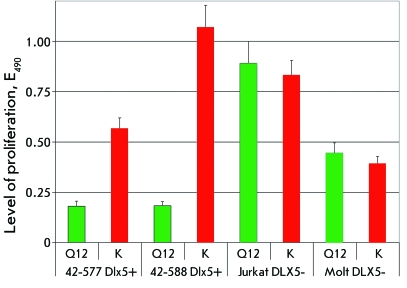
Selectivity of compound Q12 on human lymphoma cells. On the left: proliferation of Dlx5-positive mouse lymphoma cells 42-577 and 42-588. On the right: proliferation of DLX5-negative human lymphoma cells Jurkat and Molt16. Additional labeling is identical to that in *Fig. 2.*

In order to eliminate the possibility of a nonspecific impact of compounds Q12 and Q9 on cells of the lymphoid series, their action was tested on T-cell lymphoma cells of line 72 with absent expression of *Dlx5 * from Akt2-transgenic mice ( *Fig. 3* ). Cells of line 72 contain another type of chromosome rearrangement, a translocation between chromosomes 14 and 15 (t(14:15)), which results in an increased expression of protooncogene *c-myc* [ *30* ] *. Fig. 4* shows the results of the effect of compound Q12 on the proliferation of an additional two subtypes of lymphoma cells expressing *Dlx5 * (42-577 and 42-588) *,* as well as the proliferation of the human lymphoma cells Jurkat and Molt16 not expressing *DLX5* . A general conclusion can be made from the data presented in *Figs. 3 and 4 * that compounds Q9 and Q12 have no effect on the proliferation of cells not expressing *Dlx5* ; however, they are highly efficient in the suppression of the proliferation of cells in which this factor is expressed.

**Fig. 5:**
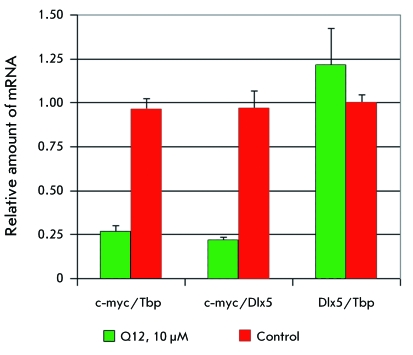
Real-time RT-PCR. The measurement of the expression of *c-myc* and *Dlx5 * in mouse lymphoma cells 42-936, after cultivation with 10 µM compound Q12 by the mRNA level. Tbp is the TATA-binding protein.

It is known that the DLX5 transcription factor can directly control the expression of protooncogene *c-myc* [8, 30]. The impact of Q12 on the expression of *c-myc * in the lymphoma cells 42-936 expressing *Dlx5* was studied by real-time RT-PCR. *Fig. 5* shows the levels of mRNA of c-myc with respect to the endogenous control, mRNA of TATA-binding protein (Tbp) or mRNA of Dlx5, as well as mRNA of Dlx5 with respect to mRNA of Tbp in the presence of 10 µM Q12 and without any addition of it. It can be seen that the expression of *c-myc* decreases considerably under the action of Q12, while the expression of *Dlx5* remains intact. These results agree with the conception of the inhibitory effect of ligand Q12 on the transcription activity of the Dlx5 factor. Although these data need to be tested on a larger number of cell lines, it is tempting to make a preliminary conclusion on the specificity of binding between the transcription factor DLX5 and ligand Q12 based on the results of this study.

The approaches used in this study made it possible to experimentally identify the most active inhibitors of Dlx5 (DLX5) out of those that were tested. Further plans include optimizing the structure of the resulting compounds in terms of parameters such as the enhancement of efficacy, reduction of possible nonspecific toxicity, and the enhancement of the metabolic stability. The next stage of this study assumes that the activity and toxicity of the optimized compounds will be assessed *in vivo, * and their *K*_d_ will be measured directly *.*

## CONCLUSIONS

With the aim of verifying the fundamental possibility of using the DLX5 transcription factor as a target for anti-tumor agents and designing drugs that can suppress the development of certain types of human tumors (T-lymphomas, lung and ovarian cancer), a search for specific ligands of the DLX5 factor was performed based on the analysis of its crystal structure. It was shown that more than 50% of compounds which were selected by docking technique are capable at micro-molar concentrations to inhibit the proliferation of mouse lymphoma cells expressing *Dlx5* . Moreover, most of the compounds active on Dlx5 positive lymphoma cells had no effect on other types of cells that do not express this transcription factor, which serves as evidence of the specificity of the selected molecules. Compounds Q12 and Q9 were found to be the best in terms of the ratio between the parameter characterizing the efficacy and the absence of nonspecific cytotoxicity. The observed decrease in the expression of *с-myc * under the action of Q12 attests to the inhibitory effect of this ligand on the transcriptional activity of the Dlx5. The compounds discovered are the first described low-molecular-weight ligands of DLX5 which can be used for subsequent chemical optimization and the development of highly efficient anti-tumor agents. 

## Abbreviations

DLX5: human transcription factor, encoded by gene*DLX5*(Distall-less homeobox gene 5)
Dlx5: mouse transcription factor encoded by gene*Dlx5*
K_d_: binding (affinity) constant
NSCLC - non-small cell lung cancer; siRNA: small interfering RNA, RT-PCR – reverse transcription polymerase chain reaction
